# Communications of the Rhode Island Medical Society for the Year 1862

**Published:** 1862-08

**Authors:** 


Communications of the Rhode Island Medical Society, for the year 1862. Published by the Society. Providence: Cook and Daniels, Printers.

This pamphlet contains the record of proceedings of the Rhode Island Medical Society, and the communications made to it, for the year ending June 4, 1862. The most important of these communications is an interesting address on "Hereditary Transmission," by B. Lincoln Ray, M.D., though all of them are worthy of a careful perusal.

